# Hair Shaft Defect in a Teenage Girl: Trichoscopy Saves the Day!

**DOI:** 10.5826/dpc.1101a80

**Published:** 2020-12-07

**Authors:** Balachandra S. Ankad, Samipa S. Mukherjee

**Affiliations:** 1 Department of Dermatology, S. Nijalingappa Medical College, Bagalkot, Karnataka, India; 2 Pediatric Dermatology, Cloudnine Hospital, Bengaluru, India

**Keywords:** trichoscopy, monilethrix, diagnosis, genotrichoses

## Introduction

Genotrichoses are difficult to diagnose and pose a diagnostic and therapeutic challenge in most cases. Accurate diagnosis of genotrichoses with hair shaft defects mandates utilization of polarized or electron microscopy. Trichoscopy, the dermoscopy of hair and scalp diseases, is a well-established diagnostic technique for common hair disorders. Recently its applications have been extended to genotrichoses [1]. Trichoscopy is useful in genotrichoses presenting with hair shaft defects such as trichorrhexis nodosa, trichorrhexis invaginata, pili annulati, and monilethrix [2]. Here we describe the importance of trichoscopy, which assisted in the diagnosis of a hair shaft disorder in a teenage girl.

## Case Presentation

**Figure 1:**
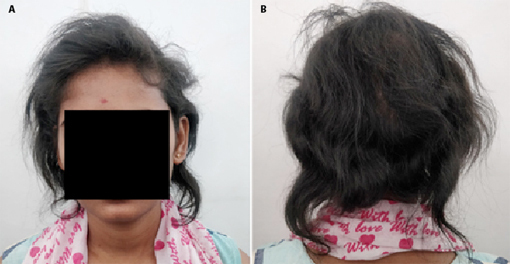
(A) Clinical image of a teenage girl with dry, lusterless, and frizzy hair on the frontal area. (B) Clinical image of the occipital area showing dry, lusterless, and frizzy hair. Note that hairs are not growing beyond the neck and shoulders.

**Figure 2:**
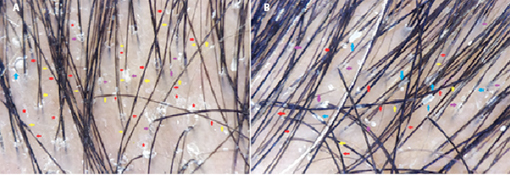
(A) Trichoscopy of monilethrix shows regular distribution of nodes (yellow arrows) and internodes (red arrows). Note that medulla is present in nodes and absent in internode segments. Regrowing hair (pigtail hair; blue arrow), perifollicular scales (purple arrows), and interfollicular scales (red stars) are well appreciated. (B) Trichoscopy of monilethrix shows regular distribution of nodes (yellow arrows) and internodes (red arrows). Hair casts (blue arrows) and perifollicular casts (purple arrows) are well appreciated.

A 17-year-old girl, born out of a non-consanguineous marriage, presented with increased hair loss, frizzy rough hair, and an inability to grow hair beyond shoulder length since childhood (Figure 1, A and B). She had received biotin supplements and 2% minoxidil topical solution in the past with no response. Examination revealed short, lusterless, fragile hair with easy breakability. A hair pull test was negative; however, the hair broke easily along its shaft. Skin, teeth and nails were normal. Systemic examination was unremarkable. Family history and past history were noncontributory. Loose anagen hair syndrome and short anagen hair syndrome were considered as differentials. Trichoscopic examination, with Illuco IDS-1000 with ×10 magnification, showed regular variations in the diameter of the hair shaft with elliptical nodes separated by internodes. Hair casts and white scales in follicular and interfollicular areas were also noted (Figure 2, A and B). Trichoscopy of eyebrows was normal. Microscopic examination was suggestive of monilethrix.

## Conclusions

Monilethrix, a rare autosomal dominant disorder with variable expression, is characterized by fragility of hair and inability to grow in length. Scalp, eyebrows, axillary, and pubic hairs are affected with associated follicular keratotic papules [2]. Affected individuals have normal hair at birth with hair changes manifest in early childhood. Clinically it presents with dull, lusterless, fragile short hairs. Trichoscopy shows regularly placed nodes and internodes along the shaft that give the characteristic beaded appearance. Elliptical nodes have normal hair diameter, medulla, and normal cortical cells, whereas internodes are narrower and are devoid of medulla with a lower number and wrinkling of cortical cells making them the site of fracture [1]. Due to multiple fractures, hair shafts are bent regularly at multiple locations with a tendency to curve in different directions producing a “regularly bended ribbon sign” [1]. A similar observation was made in this study. Furthermore, prominent scales located in the perifollicular and interfollicular areas and hair casts were noted and were attributed to the lack of proper washing by the patient because of fear of losing hairs. It should be noted here that with no family history, absence of keratotic papules, late onset of symptoms, and the presence of frizzy, lusterless, and non-growing hairs, loose anagen hair syndrome, short anagen hair syndrome and weathering of hair Nevertheless, trichoscopic examination clinched the diagnosis of monilethrix.

Although the management of monilethrix remains challenging, the use of a trichoscope for the diagnosis of this rare and unsuspicious condition is noteworthy. It is not only diagnostic, but it also helps in differentiating monilethrix from other similar conditions such as monilethrix-like hair.
